# Crystal structure of *N*-(*tert*-but­oxy­carbon­yl)phenyl­alanylde­hydro­alanine isopropyl ester (Boc–Phe–ΔAla–OiPr)

**DOI:** 10.1107/S1600536814025197

**Published:** 2014-11-29

**Authors:** Paweł Lenartowicz, Maciej Makowski, Bartosz Zarychta, Krzysztof Ejsmont

**Affiliations:** a Faculty of Chemistry, University of Opole, Oleska 48, 45-052 Opole, Poland

**Keywords:** crystal structure, de­hydro peptides, α,β-de­hydro­amino acids, de­hydro­alanine, herringbone packing

## Abstract

In the title compound, the de­hydro­dipeptide (Boc–Phe–ΔAla–OiPr, C_20_H_28_N_2_O_5_), the mol­ecule has a *trans* conformation of the *N*-methyl­amide group. The geometry of the de­hydro­alanine moiety is to some extent different from those usually found in simple peptides, indicating conjugation between the H_2_C=C group and the peptide bond. The bond angles around de­hydro­alanine have unusually high values due to the steric hindrance, the same inter­action influencing the slight distortion from planarity of the de­hydro­alanine. The mol­ecule is stabilized by intra­molecular inter­actions between the isopropyl group and the N atoms of the peptide main chain. In the crystal, an N—H⋯O hydrogen bond links the mol­ecules into ribbons, giving a herringbone head-to-head packing arrangement extending along the [100] direction. In the stacks, the mol­ecules are linked by weak C—H⋯O hydrogen-bonding associations.

## Abstract 2

In the crystal structure of the de­hydro­dipeptide (Boc-Phe-ΔAla-OiPr), the mol­ecule has a *trans* configuration of the *N*-methyl­amide group. Its geometry is different from saturated peptides but is in excellent agreement with other de­hydro­alanine compounds. In the crystal, an N—H⋯O hydrogen bond links the mol­ecules in a herringbone packing arrangement.

## Chemical context   

De­hydro­peptides are a class of compounds containing at least one residue of an α,β-de­hydro­amino acid. These compounds are of inter­est in many fields of science because of their structural and chemical properties. De­hydro­amino acids are found in natural products (Bonauer *et al.*, 2006 ▶). One of the important classes of natural bacteriocins are lanti­biotics (*e.g*. nisin, subtilin), which are biosynthesized by Gram-positive bacteria. The unsaturated amino acid is introduced into the structure of these polycyclic peptides by post-translational modification of selected serine and threonine residues (Willey & van der Donk, 2007 ▶). The development of synthetic methods for de­hydro­peptide preparation has resulted in a search for practical applications for these compounds. The de­hydro­amino acids are considered to be building blocks for the synthesis of new non-proteinogenic amino acids (Ferreira *et al.*, 2010 ▶). The double bond of the de­hydro­peptide can be used in different types of reaction, namely: addition of nucleophiles (Ferreira *et al.*, 2001 ▶); alkyl­ation, providing α,α-disubstituted amino acids (Miyabe *et al.*, 2005 ▶); Rh-catalysed conjugate addition of aryl­boronic acids providing β-aryl­alanine derivatives (Ferreira *et al.*, 2013 ▶); Cu-catalysed asymmetric hydro­boration as a step in the preparation of β-hy­droxy-α-amino acid derivatives being then used for the preparation of chiral drugs and bioactive mol­ecules (He *et al.*, 2014 ▶). Compounds containing de­hydro­amino acid residues also are considered to be inhibitors of enzymes (Makowski *et al.*, 2001 ▶; Latajka *et al.*, 2006 ▶, 2008 ▶). They are more resistant towards proteolytic enzymes than saturated analogues (English & Stammer, 1978 ▶). The presence of *sp* ^2^ hybridized carbon atoms in structures of de­hydro­peptides and the coupling of π-electrons between double and peptide bonds entail a number of structural consequences in the conformation of the peptides, and make them excellent subjects for conformational study (*e.g.* Jewgiński *et al.*, 2014 ▶, 2013 ▶; Demizu *et al.*, 2010 ▶; Lisowski *et al.*, 2008 ▶). In this paper, the preparation of the title compound, *N*-(*tert*-but­oxy­carbon­yl)-phenyl­alanylde­hydro­alanine isopropyl ester and its structure determination by single-crystal X-ray crystallographic methods are presented.
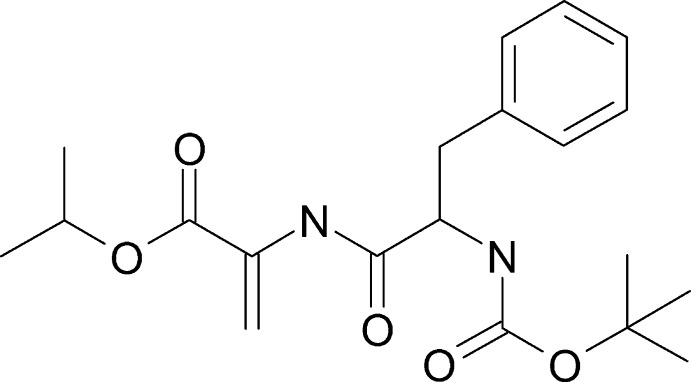


## Structural commentary   

**Figure 1:**
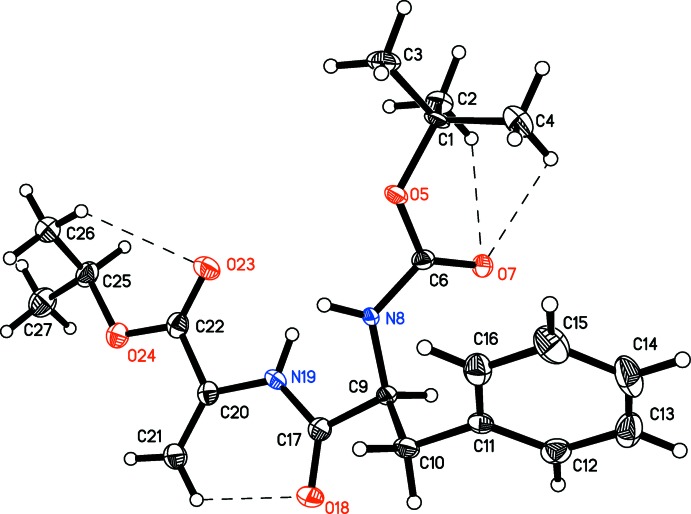
The mol­ecular structure of *N*-(*tert*-but­oxy­carbon­yl)phenyl­alanylde­hydro­alanine isopropyl ester (Boc–Phe–ΔAla–OiPr) showing 50% displacement ellipsoids. Intra­molecular C—H⋯O inter­actions are shown as dashed lines.

The mol­ecular structure of *N*-(*tert*-but­oxy­carbon­yl)phenyl­alanylde­hydro­alanine isopropyl ester (Boc–Phe–ΔAla–OiPr, C_20_H_28_N_2_O_5_) is shown in Fig. 1 ▶. The mol­ecule has a *trans*-conformation of the *N*-methyl­amide group. The geometry of the de­hydro­alanine is to some extent different from those usually found in simple peptides (Pauling, 1960 ▶). In particular, the N19—C20 bond length is shorter while C17—N19 is longer [1.402 (3) Å and 1.354 (3) Å, respectively]. This is in excellent agreement with the values reported for *N*-acetyl­dehydro­alanine (Ajó *et al.*, 1979 ▶), *N*-acetyl­bis-(de­hydro­phenyl­alanyl)glycine (Pieroni *et al.* 1975 ▶) and *N*-acetyl­ode­hydro­di­­meth­yl­amide (Rzeszotarska *et al.*, 2002 ▶) and seems to be typical for α, β-unsaturated peptide systems (Jain & Chauhan, 1996 ▶). This indicates conjugation between the H_2_C=C group and the peptide bond. The valance angles around de­hydro­alanine have unusually large values [C21—C20—N19 = 126.9 (2), C17—N19—C20 = 126.8 (2) and O18—C17—N19 = 123.5 (2)°] due to the steric hindrance between atoms C21 and O18. The same inter­action influences the slight distortion from planarity of the de­hydro­alanine moiety. The ω, ϕ and ψ torsion angles (C9—C17—N19—C20, C17—N19—C20—C22 and N19—C20—C22—O24, respectively) of the de­hydro­alanine residue are −166.9 (2), 175.1 (2) and 178.0 (2)°. The geom­etries of the phenyl­aniline and the protecting groups are normal. There are four intra­molecular C—H⋯O close contacts but three of them have a *D*—H⋯*A* angle of less than 120°.

## Supra­molecular features   

**Figure 2:**
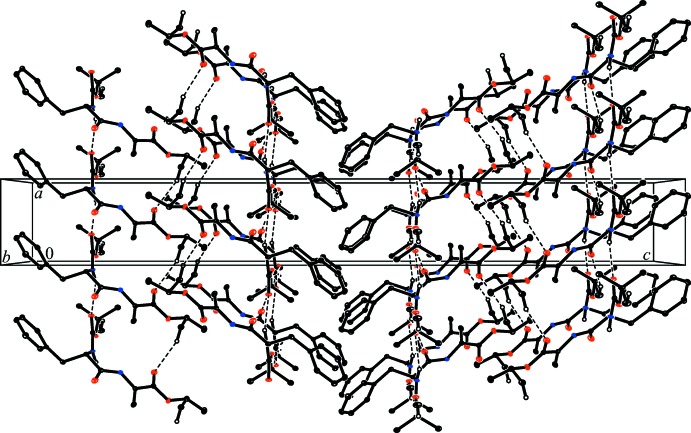
The packing diagram of the title compound, viewed along the *b* axis, showing the inter­molecular hydrogen-bonding scheme (dashed lines).

In the crystal, strong inter­molecular N8—H⋯O7^i^ hydrogen bonds (Table 1 ▶) link the mol­ecules, giving a herringbone head-to-head packing arrangement, forming ribbons which extend along [100] (Fig. 2 ▶). The ribbon structures are consolidated by weak intra-chain C—H⋯O hydrogen-bonding inter­actions.

## Synthesis and crystallization   

The de­hydro­dipeptide was obtained by condensation of *N*-protected phenyl­alanyl­amide with pyruvic acid in the presence of *p*-toluene­sulfonic acid (Makowski *et al.*, 1985 ▶). The esterification of the de­hydro­dipeptide was performed using the methodology described by Cossec *et al.* (2008 ▶). For this purpose 0.669 g (2 m*M*) of Boc–Phe–ΔAla was dissolved in 5 ml of methanol and calcium carbonate 0.329 g (1 m*M*) was added. The mixture was stirred for one h at room temperature, after which the solvent was evaporated. The residue was dissolved in 7 ml of DMF and isopropyl iodide (1.01 ml, 10 m*M*) was added in portions to the stirred mixture at room temperature during the reaction, the progress of which was monitored by thin-layer chromatography, using 5% methanol in chloro­form as eluent. After completion of the reaction, the solvent was evaporated and the oily residue was dissolved in ethyl acetate and washed consecutively with: 1 *M* HCl, saturated KHCO_3_, 0.1 *M* Na_2_S_2_O_3_ and brine. The organic layer was dried over anhydrous MgSO_4_ and the title compound was obtained in 81% yield (m.p. = 367–369 K). Recrystallization was performed using mixture of diethyl ether and hexane.

^1^H NMR (400 MHz, DMSO) δ 1.26 (*d*, *J* = 6.2 Hz, 6H, 2 × CH_3Pr^*i*^_), 1.30 (*s*, 9H, CH_3 *t*-Boc_), 2.76 (*dd*, ABX system, *J* = 13.6, 10.8 Hz, 1H, CH_*A*_H_*B* Phe_), 3.02 (*dd*, ABX system, *J* = 13.6, 3.9 Hz, 1H, CH_*A*_H_*B* Phe_), 4.27–4.39 (*m*, 1H, CH_Phe_), 5.01 (hept, *J* = 6.2 Hz, 1H, CH_Pr^*i*^_), 5.70 (*s*, 1H, C=CH_*A*_H_*B*_), 6.23 (*s*, 1H, C=CH_*A*_H_*B*_), 7.15–7.36 (*m*, 6H, ArH_Phe_ overlapped with NH_Phe_), δ 9.30 (*s*, 1H, NH_ΔAla_). ^13^C NMR (101 MHz, DMSO) δ 21.43, 28.10, 36.63, 56.34, 69.40, 78.41, 108.65, 126.29, 128.07, 129.25, 132.71, 138.03, 155.53, 162.81, 171.53. IR (KBr, cm^−1^) 3600–2800 broad (H-bonding), 1715 (C=O_ester_), 1700 (C=O_urethane_), 1690 IAB (C=O_amide_), 1632 (C=C), 1526 IIAB (C–N and N–H), 1317 (CO–N–C=and N–(C=C)–CO), 1196 and 1166 (C–O–C), 896 (=CH_2_).

## Refinement details   

Crystal data, data collection and structure refinement details are summarized in Table 2 ▶. All hydrogen atoms were positioned geometrically and treated as riding on their parent atoms with N—H = 0.88 Å and *U*_iso_ (H) = 1.2*U*_eq_(N), C—H_aromatic_ = 0.95 Å and *U*_iso_ (H) = 1.2*U*_eq_(C), C—H_meth­yl_ = 0.98 Å and *U*_iso_ (H) = 1.5*U*_eq_(C); C—H_methyl­ene_ = 0.99 Å or C—H_methine_ = 0.95 Å and *U*_iso_ (H) = 1.2*U*_eq_(C). Although not definitive, the absolute structure factor (Parsons *et al.*, 2013 ▶) with the C9(*S*) configuration, was −0.1 (6) for 1095 Friedel pairs.

## Supplementary Material

Crystal structure: contains datablock(s) global, I. DOI: 10.1107/S1600536814025197/zs2321sup1.cif

Structure factors: contains datablock(s) I. DOI: 10.1107/S1600536814025197/zs2321Isup2.hkl

Supporting information file. DOI: 10.1107/S1600536814025197/zs2321Isup3.cml

CCDC reference: 1034604

Additional supporting information:  crystallographic information; 3D view; checkCIF report

## Figures and Tables

**Table 1:** Hydrogen-bond geometry (, )

*D*H*A*	*D*H	H*A*	*D* *A*	*D*H*A*
N8H8*A*O7^i^	0.88	2.21	2.952(2)	141
C3H3*C*O18^ii^	0.98	2.51	3.423(3)	155
C21H21*A*O18	0.95	2.27	2.869(3)	120
C26H26*B*O23^i^	0.98	2.52	3.462(3)	162

Symmetry codes: (i) 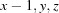; (ii) 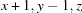.

**Table 2:** Experimental details

Crystal data	
Chemical formula	C_20_H_28_N_2_O_5_
*M*_r_	376.44
Crystal system, space group	Orthorhombic, *P*2_1_2_1_2_1_
Temperature (K)	100
*a*, *b*, *c* ()	5.2123(2), 9.5031(3), 41.3363(17)
*V* (^3^)	2047.51(13)
*Z*	4
Radiation type	Mo *K*
(mm^1^)	0.09
Crystal size (mm)	0.33 0.18 0.14

Data collection	
Diffractometer	Oxford Diffraction Xcalibur CCD
No. of measured, independent and observed [*I* > 2(*I*)] reflections	14003, 4025, 3235
*R*_int_	0.046
(sin /)_max_ (^1^)	0.617

Refinement	
*R*[*F* ^2^ > 2(*F* ^2^)], *wR*(*F* ^2^), *S*	0.046, 0.079, 0.98
No. of reflections	4025
No. of parameters	244
H-atom treatment	H-atom parameters constrained
_max_, _min_ (e ^3^)	0.22, 0.22
Absolute structure	Flack *x* determined using 1095 quotients [(*I* ^+^)(*I* )]/[(*I* ^+^)+(*I* )] (Parsons *et al.*, 2013 ▶)
Absolute structure parameter	0.1(6)

Computer programs: *CrysAlis CCD* and *CrysAlis RED* (Oxford Diffraction, 2008 ▶), *SHELXL2014* and *SHELXTL* (Sheldrick, 2008 ▶).

## supplementary crystallographic information

### Crystal data

**Table:**

C_20_H_28_N_2_O_5_	*D*_x_ = 1.221 Mg m^−^^3^
*M**_r_* = 376.44	Melting point = 367–369 K
Orthorhombic, *P*2_1_2_1_2_1_	Mo *K*α radiation, λ = 0.71073 Å
*a* = 5.2123 (2) Å	Cell parameters from 4025 reflections
*b* = 9.5031 (3) Å	θ = 3.3–26.0°
*c* = 41.3363 (17) Å	µ = 0.09 mm^−^^1^
*V* = 2047.51 (13) Å^3^	*T* = 100 K
*Z* = 4	Irregular, colourless
*F*(000) = 808	0.33 × 0.18 × 0.14 mm

### Data collection

**Table:**

Oxford Diffraction Xcalibur CCD diffractometer	3235 reflections with *I* > 2σ(*I*)
Radiation source: fine-focus sealed tube	*R*_int_ = 0.046
Graphite monochromator	θ_max_ = 26.0°, θ_min_ = 3.3°
ω scans	*h* = −3→6
14003 measured reflections	*k* = −11→11
4025 independent reflections	*l* = −50→50

### Refinement

**Table:**

Refinement on *F*^2^	Hydrogen site location: inferred from neighbouring sites
Least-squares matrix: full	H-atom parameters constrained
*R*[*F*^2^ > 2σ(*F*^2^)] = 0.046	*w* = 1/[σ^2^(*F*_o_^2^) + (0.0354*P*)^2^] where *P* = (*F*_o_^2^ + 2*F*_c_^2^)/3
*wR*(*F*^2^) = 0.079	(Δ/σ)_max_ < 0.001
*S* = 0.98	Δρ_max_ = 0.22 e Å^−^^3^
4025 reflections	Δρ_min_ = −0.22 e Å^−^^3^
244 parameters	Absolute structure: Flack *x* determined using 1095 quotients [(*I*^+^)-(*I*^-^)]/[(*I*^+^)+(*I*^-^)] (Parsons *et al.*, 2013)
0 restraints	Absolute structure parameter: −0.1 (6)

### Special details

**Table:**

Geometry. All esds (except the esd in the dihedral angle between two l.s. planes) are estimated using the full covariance matrix. The cell esds are taken into account individually in the estimation of esds in distances, angles and torsion angles; correlations between esds in cell parameters are only used when they are defined by crystal symmetry. An approximate (isotropic) treatment of cell esds is used for estimating esds involving l.s. planes.

### Fractional atomic coordinates and isotropic or equivalent isotropic displacement parameters (Å^2^)

**Table:**

	*x*	*y*	*z*	*U*_iso_*/*U*_eq_	
C1	1.2709 (4)	0.0969 (2)	0.10948 (7)	0.0184 (6)	
C2	1.4195 (5)	0.1063 (3)	0.14083 (7)	0.0249 (7)	
H2A	1.5204	0.1933	0.1411	0.037*	
H2B	1.5348	0.0252	0.1427	0.037*	
H2C	1.2995	0.1065	0.1591	0.037*	
C3	1.1120 (5)	−0.0366 (3)	0.10835 (8)	0.0325 (7)	
H3A	1.0176	−0.0406	0.0879	0.049*	
H3B	0.9903	−0.0369	0.1264	0.049*	
H3C	1.2254	−0.1185	0.1100	0.049*	
C4	1.4374 (5)	0.1068 (3)	0.07954 (7)	0.0264 (7)	
H4A	1.5397	0.1932	0.0804	0.040*	
H4B	1.3282	0.1085	0.0602	0.040*	
H4C	1.5520	0.0252	0.0786	0.040*	
O5	1.0713 (3)	0.20736 (16)	0.10856 (4)	0.0194 (4)	
C6	1.1322 (5)	0.3446 (2)	0.10987 (6)	0.0151 (5)	
O7	1.3475 (3)	0.39442 (16)	0.10970 (4)	0.0188 (4)	
N8	0.9116 (3)	0.4203 (2)	0.11214 (5)	0.0148 (5)	
H8A	0.7675	0.3751	0.1160	0.018*	
C9	0.9025 (4)	0.5718 (2)	0.10849 (6)	0.0150 (5)	
H9A	1.0784	0.6105	0.1122	0.018*	
C10	0.8141 (5)	0.6145 (3)	0.07443 (6)	0.0176 (6)	
H10A	0.6402	0.5761	0.0707	0.021*	
H10B	0.8020	0.7184	0.0734	0.021*	
C11	0.9882 (5)	0.5648 (3)	0.04781 (6)	0.0167 (6)	
C12	1.1944 (5)	0.6462 (3)	0.03752 (6)	0.0231 (6)	
H12A	1.2281	0.7335	0.0479	0.028*	
C13	1.3509 (5)	0.6026 (3)	0.01250 (7)	0.0312 (7)	
H13A	1.4897	0.6601	0.0056	0.037*	
C14	1.3057 (6)	0.4757 (3)	−0.00244 (7)	0.0338 (8)	
H14A	1.4134	0.4455	−0.0196	0.041*	
C15	1.1046 (5)	0.3924 (3)	0.00748 (7)	0.0317 (7)	
H15A	1.0739	0.3046	−0.0028	0.038*	
C16	0.9473 (5)	0.4367 (3)	0.03241 (6)	0.0244 (7)	
H16A	0.8088	0.3787	0.0391	0.029*	
C17	0.7185 (5)	0.6377 (3)	0.13324 (6)	0.0177 (6)	
O18	0.6189 (4)	0.75177 (19)	0.12844 (4)	0.0266 (5)	
N19	0.6785 (4)	0.5605 (2)	0.16029 (5)	0.0168 (5)	
H19A	0.7841	0.4897	0.1636	0.020*	
C20	0.4865 (4)	0.5814 (3)	0.18356 (6)	0.0163 (6)	
C21	0.3165 (5)	0.6849 (3)	0.18436 (6)	0.0233 (6)	
H21A	0.3171	0.7549	0.1679	0.028*	
H21B	0.1940	0.6892	0.2013	0.028*	
C22	0.4947 (5)	0.4660 (3)	0.20809 (6)	0.0197 (6)	
O23	0.6457 (4)	0.36963 (19)	0.20637 (4)	0.0282 (5)	
O24	0.3215 (3)	0.48235 (17)	0.23152 (4)	0.0232 (4)	
C25	0.3210 (5)	0.3753 (3)	0.25720 (6)	0.0249 (6)	
H25A	0.5018	0.3520	0.2633	0.030*	
C26	0.1889 (6)	0.2446 (3)	0.24504 (6)	0.0274 (7)	
H26A	0.2849	0.2060	0.2267	0.041*	
H26B	0.0143	0.2682	0.2381	0.041*	
H26C	0.1814	0.1746	0.2624	0.041*	
C27	0.1881 (6)	0.4430 (3)	0.28559 (6)	0.0323 (7)	
H27A	0.2836	0.5269	0.2923	0.049*	
H27B	0.1807	0.3761	0.3036	0.049*	
H27C	0.0135	0.4699	0.2793	0.049*	

### Atomic displacement parameters (Å^2^)

**Table:**

	*U*^11^	*U*^22^	*U*^33^	*U*^12^	*U*^13^	*U*^23^
C1	0.0114 (11)	0.0108 (13)	0.0330 (15)	0.0059 (10)	−0.0014 (12)	−0.0004 (12)
C2	0.0222 (14)	0.0209 (15)	0.0315 (16)	0.0048 (13)	0.0001 (13)	0.0041 (13)
C3	0.0235 (14)	0.0132 (13)	0.061 (2)	0.0028 (12)	−0.0025 (16)	0.0000 (14)
C4	0.0188 (14)	0.0302 (17)	0.0301 (16)	0.0049 (14)	−0.0019 (12)	−0.0077 (14)
O5	0.0107 (9)	0.0111 (9)	0.0364 (11)	0.0019 (7)	0.0009 (9)	−0.0001 (8)
C6	0.0163 (13)	0.0113 (12)	0.0177 (14)	−0.0004 (11)	−0.0003 (12)	−0.0001 (11)
O7	0.0097 (8)	0.0159 (9)	0.0308 (10)	−0.0025 (8)	0.0001 (8)	0.0022 (8)
N8	0.0084 (9)	0.0100 (10)	0.0261 (12)	0.0007 (8)	0.0041 (9)	0.0023 (10)
C9	0.0126 (11)	0.0106 (12)	0.0218 (14)	−0.0004 (10)	0.0007 (11)	0.0011 (12)
C10	0.0163 (12)	0.0147 (13)	0.0218 (14)	0.0026 (12)	−0.0026 (12)	0.0014 (11)
C11	0.0146 (12)	0.0189 (14)	0.0167 (13)	0.0044 (12)	−0.0036 (11)	0.0053 (12)
C12	0.0219 (14)	0.0228 (15)	0.0246 (15)	0.0011 (14)	−0.0054 (14)	0.0055 (12)
C13	0.0203 (14)	0.047 (2)	0.0261 (16)	0.0016 (16)	0.0003 (14)	0.0160 (15)
C14	0.0245 (15)	0.056 (2)	0.0208 (16)	0.0117 (17)	0.0058 (13)	0.0011 (15)
C15	0.0312 (16)	0.0375 (19)	0.0265 (16)	0.0061 (16)	0.0001 (14)	−0.0093 (14)
C16	0.0197 (14)	0.0281 (16)	0.0254 (16)	−0.0011 (13)	0.0002 (12)	0.0002 (13)
C17	0.0154 (13)	0.0148 (14)	0.0228 (15)	−0.0031 (12)	−0.0022 (12)	−0.0021 (12)
O18	0.0284 (11)	0.0159 (10)	0.0356 (12)	0.0083 (9)	0.0094 (9)	0.0026 (9)
N19	0.0135 (10)	0.0170 (11)	0.0198 (12)	0.0047 (10)	0.0000 (9)	0.0016 (10)
C20	0.0149 (12)	0.0171 (14)	0.0168 (13)	−0.0033 (12)	−0.0017 (11)	−0.0029 (12)
C21	0.0232 (14)	0.0245 (15)	0.0221 (15)	0.0037 (13)	0.0067 (14)	0.0011 (12)
C22	0.0165 (13)	0.0235 (16)	0.0193 (14)	−0.0037 (12)	−0.0031 (12)	0.0003 (12)
O23	0.0242 (10)	0.0282 (11)	0.0322 (11)	0.0096 (10)	0.0027 (10)	0.0085 (9)
O24	0.0231 (10)	0.0256 (11)	0.0207 (10)	0.0009 (9)	0.0058 (9)	0.0035 (8)
C25	0.0228 (14)	0.0280 (15)	0.0238 (15)	−0.0029 (15)	0.0007 (13)	0.0102 (13)
C26	0.0238 (15)	0.0253 (15)	0.0333 (16)	0.0004 (14)	0.0023 (14)	0.0057 (13)
C27	0.0368 (17)	0.0341 (18)	0.0262 (16)	−0.0036 (16)	0.0039 (14)	0.0025 (14)

### Geometric parameters (Å, º)

**Table:**

C1—O5	1.478 (3)	C13—C14	1.375 (4)
C1—C2	1.513 (4)	C13—H13A	0.9500
C1—C4	1.514 (4)	C14—C15	1.376 (4)
C1—C3	1.516 (3)	C14—H14A	0.9500
C2—H2A	0.9800	C15—C16	1.382 (4)
C2—H2B	0.9800	C15—H15A	0.9500
C2—H2C	0.9800	C16—H16A	0.9500
C3—H3A	0.9800	C17—O18	1.218 (3)
C3—H3B	0.9800	C17—N19	1.354 (3)
C3—H3C	0.9800	N19—C20	1.402 (3)
C4—H4A	0.9800	N19—H19A	0.8800
C4—H4B	0.9800	C20—C21	1.324 (3)
C4—H4C	0.9800	C20—C22	1.494 (3)
O5—C6	1.343 (3)	C21—H21A	0.9500
C6—O7	1.218 (3)	C21—H21B	0.9500
C6—N8	1.360 (3)	C22—O23	1.209 (3)
N8—C9	1.448 (3)	C22—O24	1.333 (3)
N8—H8A	0.8800	O24—C25	1.470 (3)
C9—C10	1.536 (3)	C25—C26	1.506 (4)
C9—C17	1.536 (3)	C25—C27	1.507 (4)
C9—H9A	1.0000	C25—H25A	1.0000
C10—C11	1.503 (3)	C26—H26A	0.9800
C10—H10A	0.9900	C26—H26B	0.9800
C10—H10B	0.9900	C26—H26C	0.9800
C11—C16	1.390 (4)	C27—H27A	0.9800
C11—C12	1.391 (3)	C27—H27B	0.9800
C12—C13	1.381 (4)	C27—H27C	0.9800
C12—H12A	0.9500		
			
O5—C1—C2	109.9 (2)	C11—C12—H12A	119.4
O5—C1—C4	109.7 (2)	C14—C13—C12	119.9 (3)
C2—C1—C4	113.8 (2)	C14—C13—H13A	120.0
O5—C1—C3	102.06 (17)	C12—C13—H13A	120.0
C2—C1—C3	110.8 (2)	C13—C14—C15	120.1 (3)
C4—C1—C3	109.9 (2)	C13—C14—H14A	120.0
C1—C2—H2A	109.5	C15—C14—H14A	120.0
C1—C2—H2B	109.5	C14—C15—C16	119.9 (3)
H2A—C2—H2B	109.5	C14—C15—H15A	120.0
C1—C2—H2C	109.5	C16—C15—H15A	120.0
H2A—C2—H2C	109.5	C15—C16—C11	121.1 (3)
H2B—C2—H2C	109.5	C15—C16—H16A	119.4
C1—C3—H3A	109.5	C11—C16—H16A	119.4
C1—C3—H3B	109.5	O18—C17—N19	123.5 (2)
H3A—C3—H3B	109.5	O18—C17—C9	121.4 (2)
C1—C3—H3C	109.5	N19—C17—C9	115.1 (2)
H3A—C3—H3C	109.5	C17—N19—C20	126.8 (2)
H3B—C3—H3C	109.5	C17—N19—H19A	116.6
C1—C4—H4A	109.5	C20—N19—H19A	116.6
C1—C4—H4B	109.5	C21—C20—N19	126.9 (2)
H4A—C4—H4B	109.5	C21—C20—C22	123.2 (2)
C1—C4—H4C	109.5	N19—C20—C22	109.9 (2)
H4A—C4—H4C	109.5	C20—C21—H21A	120.0
H4B—C4—H4C	109.5	C20—C21—H21B	120.0
C6—O5—C1	121.48 (18)	H21A—C21—H21B	120.0
O7—C6—O5	126.5 (2)	O23—C22—O24	124.9 (2)
O7—C6—N8	125.0 (2)	O23—C22—C20	122.3 (2)
O5—C6—N8	108.46 (19)	O24—C22—C20	112.8 (2)
C6—N8—C9	123.12 (19)	C22—O24—C25	116.4 (2)
C6—N8—H8A	118.4	O24—C25—C26	109.3 (2)
C9—N8—H8A	118.4	O24—C25—C27	105.5 (2)
N8—C9—C10	111.61 (19)	C26—C25—C27	113.7 (2)
N8—C9—C17	110.89 (19)	O24—C25—H25A	109.4
C10—C9—C17	108.39 (19)	C26—C25—H25A	109.4
N8—C9—H9A	108.6	C27—C25—H25A	109.4
C10—C9—H9A	108.6	C25—C26—H26A	109.5
C17—C9—H9A	108.6	C25—C26—H26B	109.5
C11—C10—C9	114.0 (2)	H26A—C26—H26B	109.5
C11—C10—H10A	108.7	C25—C26—H26C	109.5
C9—C10—H10A	108.7	H26A—C26—H26C	109.5
C11—C10—H10B	108.7	H26B—C26—H26C	109.5
C9—C10—H10B	108.7	C25—C27—H27A	109.5
H10A—C10—H10B	107.6	C25—C27—H27B	109.5
C16—C11—C12	117.8 (2)	H27A—C27—H27B	109.5
C16—C11—C10	121.2 (2)	C25—C27—H27C	109.5
C12—C11—C10	121.0 (2)	H27A—C27—H27C	109.5
C13—C12—C11	121.2 (3)	H27B—C27—H27C	109.5
C13—C12—H12A	119.4		
			
C2—C1—O5—C6	60.9 (3)	C12—C11—C16—C15	−0.6 (4)
C4—C1—O5—C6	−65.0 (3)	C10—C11—C16—C15	178.7 (2)
C3—C1—O5—C6	178.5 (2)	N8—C9—C17—O18	−156.2 (2)
C1—O5—C6—O7	5.3 (4)	C10—C9—C17—O18	−33.4 (3)
C1—O5—C6—N8	−173.4 (2)	N8—C9—C17—N19	24.2 (3)
O7—C6—N8—C9	12.2 (4)	C10—C9—C17—N19	147.0 (2)
O5—C6—N8—C9	−169.1 (2)	O18—C17—N19—C20	13.5 (4)
C6—N8—C9—C10	99.5 (3)	C9—C17—N19—C20	−166.9 (2)
C6—N8—C9—C17	−139.5 (2)	C17—N19—C20—C21	−3.8 (4)
N8—C9—C10—C11	−61.4 (3)	C17—N19—C20—C22	175.1 (2)
C17—C9—C10—C11	176.2 (2)	C21—C20—C22—O23	177.4 (2)
C9—C10—C11—C16	91.7 (3)	N19—C20—C22—O23	−1.5 (3)
C9—C10—C11—C12	−89.0 (3)	C21—C20—C22—O24	−3.0 (3)
C16—C11—C12—C13	1.0 (4)	N19—C20—C22—O24	178.0 (2)
C10—C11—C12—C13	−178.3 (2)	O23—C22—O24—C25	1.1 (4)
C11—C12—C13—C14	−0.7 (4)	C20—C22—O24—C25	−178.4 (2)
C12—C13—C14—C15	0.1 (4)	C22—O24—C25—C26	−78.0 (3)
C13—C14—C15—C16	0.3 (4)	C22—O24—C25—C27	159.4 (2)
C14—C15—C16—C11	0.0 (4)		

### Hydrogen-bond geometry (Å, º)

**Table:**

*D*—H···*A*	*D*—H	H···*A*	*D*···*A*	*D*—H···*A*
N8—H8*A*···O7^i^	0.88	2.21	2.952 (2)	141
C2—H2*A*···O7	0.98	2.48	3.049 (3)	117
C3—H3*C*···O18^ii^	0.98	2.51	3.423 (3)	155
C4—H4*A*···O7	0.98	2.47	3.040 (3)	116
C21—H21*A*···O18	0.95	2.27	2.869 (3)	120
C26—H26*A*···O23	0.98	2.58	3.104 (3)	114
C26—H26*B*···O23^i^	0.98	2.52	3.462 (3)	162

Symmetry codes: (i) *x*−1, *y*, *z*; (ii) *x*+1, *y*−1, *z*.
